# Black blood vessel wall MRI to identify vulnerable atherosclerotic plaque in a non-stenotic intracranial vertebral artery as a cause of acute ischaemia

**DOI:** 10.1259/bjrcr.20200061

**Published:** 2020-06-18

**Authors:** Sundip Dhanvant Udani, Pervinder Bhogal

**Affiliations:** 1 Department of Neuroradiology, The Royal London Hospital, Whitechapel Road, London, E1 1BB, United Kingdom; 2 Department of Interventional Neuroradiology, The Royal London Hospital, Whitechapel Road, London, E1 1BB, United Kingdom

## Abstract

Conventional neuroimaging techniques for investigating the cause of stroke are mainly centred on investigating luminal stenosis. The pathophysiology of intracranial atherosclerotic disease (ICAD) and stroke is complex and extends beyond just vessel narrowing. The concept of the vulnerable atherosclerotic plaque, that can result in acute coronary syndromes, has been well described in the cardiac literature^1,2^although this concept is less well accepted among stroke physicians. We describe a case of a 61-year-old male with acute neurological sequelae from a non-stenotic atherosclerotic plaque of the intracranial vertebral artery. This case report describes the additional use of vessel wall MRI techniques to aid the radiologist in identifying such vulnerable lesions and therefore helping to tailor management and prevent further clinical deterioration.

## Case presentation

A 61-year-old male presented to the emergency department with a 2-h history of new alexia, intermittent expressive dysphasia and right upper limb ataxia. He had a National Institutes for Health Stroke Scale (NIHSS) score of 6 and modified Rankin Score (mRS) for neurological disability of 4. His vision assessed by confrontation remained intact. He had a past medical history of diet controlled type 2 diabetes mellitus and hypertension controlled with Ramipril and Amlodipine 5 mg.

## Investigations

Initial base line parameters including full blood count, urea and electrolytes, CRP, chest X-ray and ECG were all normal. Doppler, echocardiogram and a further 24-h ECG were normal.

Non-contrast CT demonstrated foci of low attenuation in the right superomedial cerebellar hemisphere and left occipital lobe suggestive of acute ischaemia. The CTA revealed that only the right vertebral artery (VA) contributed to the basilar artery. There was no intra-arterial thrombus or evidence of stenotic atherosclerotic disease especially proximally along the VA.

**Figure 1.**
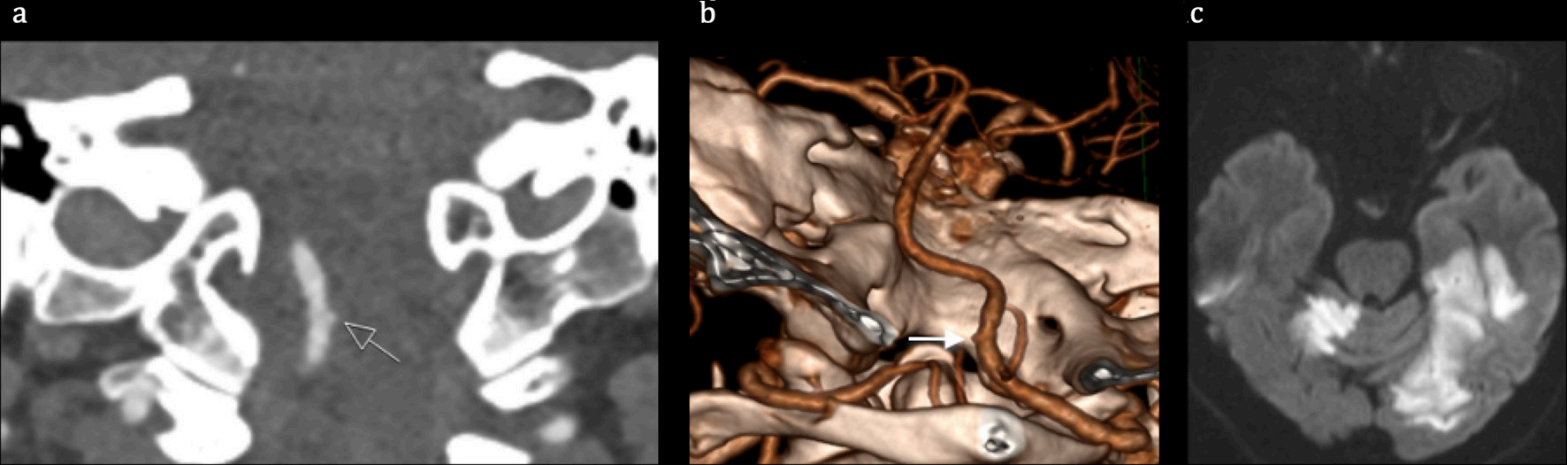
Neuroimaging (CTA, Diffusion Weighted MRI) following acute neurological symptoms. Coronal (a) and 3D posteroanterior CTA (b) demonstrating subtle irregularity of the right V4 VA. DWI demonstrating acute ischaemic lesions in the right cerebellum, left parahippocampal and fusiform gyri and left occipital lobe (c).

A very subtle irregularity, thought to represent a non-stenotic ulcerated plaque, was seen on the right V4 segment VA (Figure 1). The MRI confirmed restricted diffusion in the right cerebellar hemisphere, left parahippocampal gyrus, fusiform gyrus and left occipital lobe (Figure 1).

**Figure 2.**
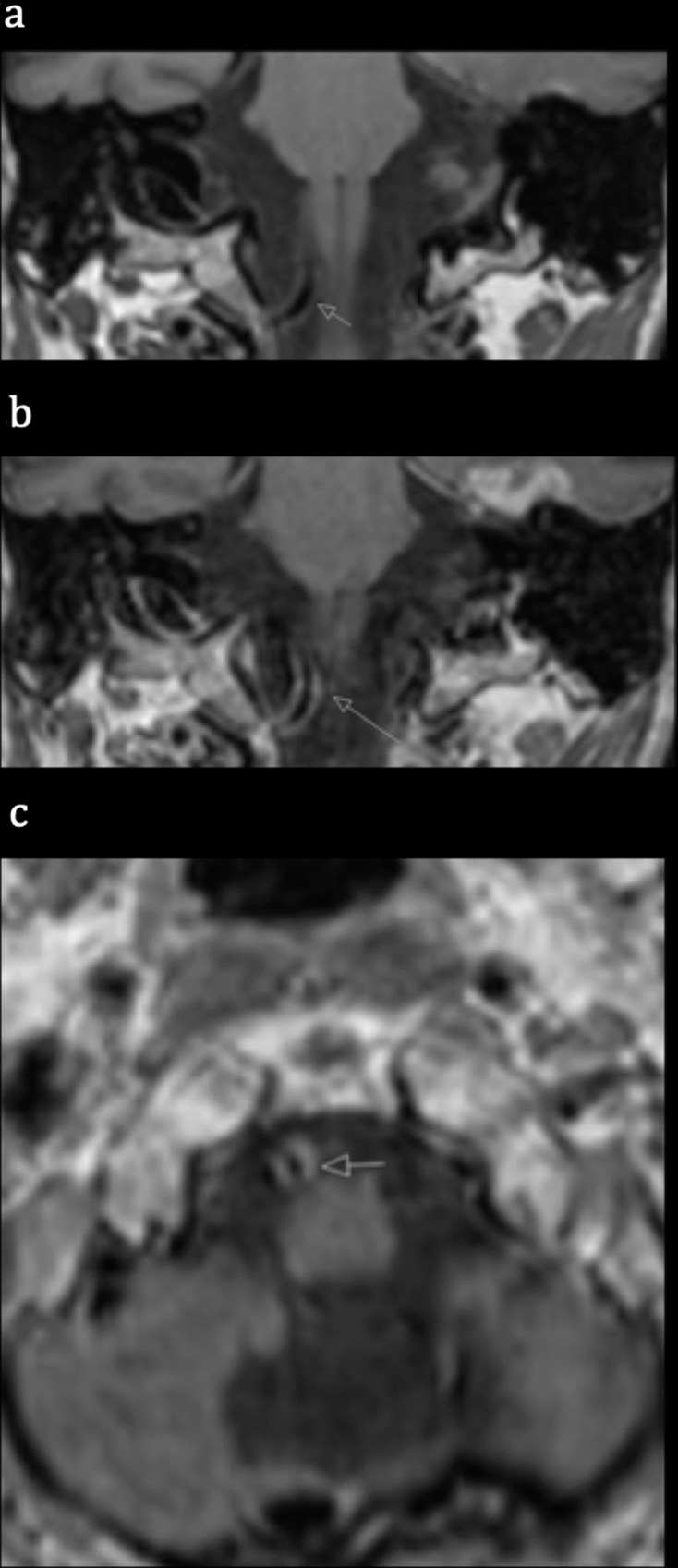
Blood suppressed vessel wall MRI coronal T1W pre-contrast (a), coronal T1W (b) and axial T1W (c) post-gadolinium demonstrating eccentric enhancement of the medial wall of the right V4 segment VA.

Blood suppressed vessel wall MRI demonstrated circumferential enhancement at the site of the plaque that was slightly eccentric with greater enhancement seen medially (Figure 2). This correlated with the subtle abnormality on CTA. The longer segment of peri-plaque enhancement is due to the diseased vessel rather than a long segment atherosclerotic plaque. No abnormal intracranial enhancement was seen elsewhere to suggest either extensive intracranial atherosclerotic disease (ICAD) or vasculitis. As the CRP and ESR (C-reactive protein and erythrocyte sedimentation rate) were within normal limits, this would also make a vasculitis unlikely. A sequelae of vessel dissection is also not favoured as this would be an atypical appearance, an unusual location and neither is there evidence of vessel narrowing or pseudoaneurysm formation.

## Treatment

The patient was treated with dual anti-platelets, clopidogrel (600 mg loading, 75 mg OD) and aspirin (75 mg OD). At 90 days follow-up, there was a residual deficit of an expressive dysphasia, with a NIHSS and mRS of 3. He had not suffered any further acute neurological insult.

## Discussion

Stroke is the second most common cause of death and third most common cause of disability worldwide.^3^ The rate of first time strokes in people aged 45 and over in the UK is expected to increase by 59% by 2035.^4^ The number of stroke survivors aged 45 and over living in the UK is expected to rise by 123%.^4^

The risk factors for stroke are similar for coronary artery disease. Strategies to treat hypertension, hypercholesterolaemia and diabetes as well as address factors such as exercise, smoking, diet and abdominal obesity have been effective in reducing stroke mortality.^5^

ICAD is the most common cause of ischaemic stroke worldwide.^6^ Racial and ethnic differences have been observed with increased prevalence of ICAD causing stroke in Asian (30–50%) and black (15–29%) populations compared to the white population (5–10%).^7^

Three main mechanisms of stroke related to ICAD have been hypothesised. Hypoperfusion distal to a highly stenotic artery can result in water-shed type infarctions. Artery-to-artery embolism from a ruptured plaque and thrombo-occlusion of the diseased artery, which are associated with wedge-shaped territorial patterns of infarct usually larger than 1.5 cm.^8^ Branch atheromatous disease typically results in subcortical perforating pattern of infarcts less than 1.5 cm secondary to plaque extension over small penetrating artery ostia. Mixed patterns are commonly observed for example when hypoperfusion prevents appropriate washout of distal emboli.

Common sites for ICAD include the internal carotid artery after it enters the petrosal bone, middle cerebral artery and basilar artery with the intracranial vertebral and posterior cerebral arteries less commonly affected.^9^ Imaging plays a fundamental role in the diagnosis and management of patients with thromboembolic stroke. Luminal stenosis, as measured by conventional imaging techniques, remains the principle means for determining treatment decisions in patients with intracranial stenotic disease. Although the risk of stroke is highest with severe stenosis (>70%), patients with moderate stenosis (50–69%) also suffer from recurrent ischaemic stroke.^10^ The degree of stenosis thus forms only part of the picture and as such does not really capture the entire essence of the underlying process.

Atherosclerosis is a chronic disorder characterized by inflammation and dysfunction of the arterial intima due to excessive cholesterol deposition. Hypercholesterolaemia is one of the major modifiable risk factors and key initiators of atherosclerosis. These risk factors are associated with increased endothelial permeability and increased low-density lipoprotein cholesterol migration into the subendothelial space. Together with intimal smooth muscle cell proliferation and collagen production, foam cells are responsible for the formation of the fatty streaks that are characteristic of an atherosclerotic plaque.^11^

Atherosclerotic plaques may be stable or unstable. Stable plaques are characterized by a slow growing mature plaque with concurrent luminal stenosis. The unstable plaques are defined by rapid lipid deposition and the formation of a thin fibrous cap that is highly susceptible to rupture and thrombosis.^11^

The concept of the vulnerable atherosclerotic plaque is well described in the cardiac literature. The histological and imaging data have consistently demonstrated that culprit plaques responsible for myocardial infarction have the following characteristics—a large plaque volume and lipid necrotic core, positive remodeling (outward bulging) of the outer surface of the artery, peripheral neovascularization, a thin fibrous cap, microcalcification, intra plaque haemorrhage and chronic inflammation.^12^

As well as being associated with acute coronary syndrome and sudden death, vulnerable atherosclerotic plaques are responsible for thromboembolic events in carotid and lower limb vessels.^13^ It has also been well documented in the literature that anterior cerebral circulation infarcts correlate well with atherosclerotic plaque vulnerability of the cervical carotid artery in addition to its degree of stenosis.^14,15^ Carotid plaque ulceration is a key feature associated with plaque vulnerability and is an indicator of previous plaque rupture thereby exposing thrombogenic substrate to circulating blood and triggering platelet aggregation and the coagulation cascade. It therefore represents an increased risk for new and possible future cerebrovascular insults.

Histological studies have confirmed that a thin fibrous cap and large lipid core can predispose to the development of ischaemic disease.^16^ A thick fibrous cap reduces the circumferential tensile stress and prevents contact between the lipid-rich necrotic core and the blood whereas a thinner fibrous cap is more likely to experience tensile stress and is predisposed to rupture. A cross-sectional study demonstrated that patients with ruptured fibrous caps were 23 times more likely to have TIA (Transient Ischaemic Attack) or stroke compared to patients with thick fibrous caps.^17^

In a study of 41 patients with symptomatic carotid artery stenosis (>70%) using black-blood MRI techniques, the number of centrum semiovale infarcts were significantly higher (*p* = 0.04) in patients with a lipid-core plaque compared to those without.^14^ This was significant even after adjustment for severity of carotid stenosis. It thus stands to reason that the ability to be able to identify subclinical vulnerable plaques could enable clinicians to effectively tailor therapeutic decision-making in this subgroup of patients.

**Figure 3.**
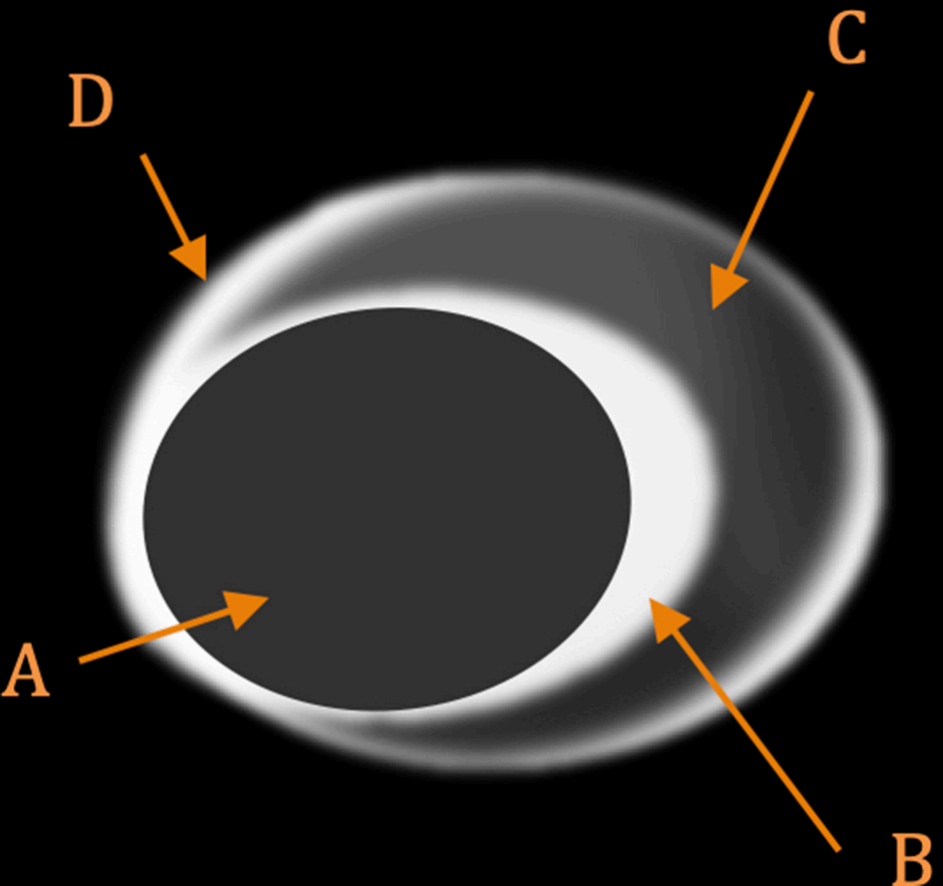
Cross-sectional illustration through an atherosclerotic arterial plaque. ‘A’ represents the arterial lumen, ‘B’ the enhancing fibrous cap, ‘C’ the non-enhancing lipid core and ‘D’ increased vasa vasorum in the adventitia of the artery.

The cause of atherosclerotic plaque enhancement is not known but is thought to be secondary to a combination of local inflammation, neovascularization and plaque infiltration of the vasa vasorum.^18,19^Vessel wall MRI appearance of carotid atherosclerotic plaques which have had corresponding carotid endarterectomy specimen analysis, provided evidence for the enhancing layer adjacent to the lumen to represent the fibrous cap, the non-enhancing adjacent layer to represent the lipid core and the peripheral thin rim of enhancement secondary to increased vasa vasorum in the adventitia of the artery^20^ (Figure 3).

Intracranial vessel wall imaging may prove a useful adjunct to conventional imaging to identify symptomatic, non-stenotic disease of the intracranial arteries. Wang et al^21^ recently performed a systematic review to investigate the clinical significance of ICAD without significant stenosis (<50%) by high-resolution VWMRI. They identified 21 studies with 463 patients with no stenosis and 651 patients with <50% stenosis. Of the 463 patients with acute stroke aetiologies attributed to non-ICAD causes and with no evidence of ICAD on time-of-flight MRA, VWMRI demonstrated ICAD in 233 patients (50.6%, 95% CI, 46.1%, 55.1%, fixed effect model). Among the 651 acute ischaemic stroke patients who had aetiologies other than ICAD excluded and had intracranial plaques identified by VWMRI, 321 (51.2%, 95% CI, 38.4%,64.0%, random effects model) had MRA results revealing <50% stenosis. Interestingly, the identification of plaques in non-stenotic arteries was associated with larger infarction,^22^ progressive motor dysfunction^23^ and worse functional outcome.^24^ This may be explained by a failure to recognise ICAD as the potential cause of the stroke and subsequently adopting an inappropriate treatment pathway.

Our case highlights the effective use of vessel wall MRI to identify a non-stenotic ulcerated atherosclerotic plaque as cause of an acute neurological event in the posterior circulation. Black-blood vessel wall MRI enabled us to determine the site of disease where there was no evidence of a significant stenosis. We were able to demonstrate an eccentric focus of enhancement of the medial wall of the V4 segment of the VA thought to represent the site of ruptured fibrous cap. This is on a background of more concentric VA enhancement thought to represent increased vasa vasorum, and which importantly must not be mistaken for vasculitis.

Interestingly, the location of enhancement has also been shown to affect clinical outcome. Lou et al used quantitative contrast-enhanced 3T high-resolution MR techniques and demonstrated that those patients with recent infarction had greater vessel wall enhancement proximal to the maximal luminal narrowing of the basilar artery (*p* = 0.046).^25^ They also demonstrated a statistical significance in vessel wall enhancement proximal to the stenosis in patients who went on to later develop subsequent ischaemic insults (*p* = 0.014).^25^ A similar situation is also seen within the cardiac literature. In a study on coronary atherosclerosis, plaque rupture was more frequently seen at the proximal, upstream side-of maximal stenosis and is thought that this is because it is the region most exposed to higher wall shear stress.^26^ Thus it is important to appreciate that vessel wall enhancement is not equally distributed along the course of the atherosclerotic vessel and proximal enhancement is associated with higher rate of both recent infarction and subsequent ischaemic stroke.^25^

A recent study of 25 unruptured intracranial aneurysms using 7T VWMRI demonstrated parent arteries exhibiting higher contrast enhancement in regions closer to the aneurysm neck.^27^ This is likely due to an inflammatory vasculopathic process that can ultimately result in aneurysm formation. Therefore VWMRI may have the potential for detecting early disease prior to any structural deficit being present.

Currently CT provides a surrogate marker of atherosclerotic plaque burden and together with ultrasound can help assess percentage stenosis in medium-sized and large-sized vessels. As our case highlights, this is not the complete picture as we describe a patient without significant stenosis or generalised atherosclerotic disease burden. Understanding plaque biology is key. Being able to image the pathophysiological process involved in addition to vessel morphology offers exciting new insights in understanding patient diagnosis and thus being able to aid in effectively tailoring clinical management.

## Learning points

1. The case presented the effective use of black-blood VWMRI imaging to corroborate a subtle finding on CTA and identify a high-risk vulnerable atherosclerotic plaque within the intracranial vertebral artery.
2. VWMRI identifies a culprit plaque in approximately 50% of patients with no evidence of stenosis or non-significant stenosis (<50%).
3. Vulnerable atherosclerotic plaques demonstrate chronic inflammation, a central lipid core, a thin fibrous cap, intra-plaque haemorrhage, microcalcification and peripheral neovascularization.
4. Focal eccentric enhancement of the vessel wall can identify the site of fibrin cap rupture of dangerous plaque.
5. The relationship of vessel wall enhancement to focal stenosis (*i.e.* more proximal) can help identify patients who are at increased risk of developing subsequent ischaemia.
6. The combination of imaging modalities allowed the identification of a subtle lesion that is a strong predictor for possible future cerebrovascular events, which therefore aided in tailoring more aggressive clinical management.
